# AhR activation underlies the CYP1A autoinduction by A-998679 in rats

**DOI:** 10.3389/fgene.2012.00213

**Published:** 2012-10-26

**Authors:** Michael J. Liguori, Chih-Hung Lee, Hong Liu, Rita Ciurlionis, Amy C. Ditewig, Stella Doktor, Mark E. Andracki, Gerard D. Gagne, Jeffrey F. Waring, Kennan C. Marsh, Murali Gopalakrishnan, Eric A. G. Blomme, Yi Yang

**Affiliations:** ^1^Abbott Laboratories, Department of Cellular, Molecular, and Exploratory ToxicologyAbbott Park, IL, USA; ^2^Abbott Laboratories, Neuroscience ResearchAbbott Park, IL, USA; ^3^Abbott Laboratories, Drug Metabolism and PharmacokineticsAbbott Park, IL, USA

**Keywords:** aryl hydrocarbon receptor, cytochrome P450, autoinduction

## Abstract

Xenobiotic-mediated induction of cytochrome P450 (CYP) drug metabolizing enzymes (DMEs) is frequently encountered in drug discovery and can influence disposition, pharmacokinetic, and toxicity profiles. The CYP1A subfamily of DMEs plays a central role in the biotransformation of several drugs and environmental chemicals. Autoinduction of drugs through CYP3A enzymes is a common mechanism for their enhanced clearance. However, autoinduction via CYP1A is encountered less frequently. In this report, an experimental compound, A-998679 [3-(5-pyridin-3-yl-1,2,4-oxadiazol-3-yl) benzonitrile], was shown to enhance its own clearance via induction of Cyp1a1 and Cyp1a2. Rats were dosed for 5 days with 30, 100, and 200 mg/kg/day A-998679. During the dosing period, the compound's plasma AUC decreased at 30 mg/kg (95%) and 100 mg/kg (80%). Gene expression analysis and immunohistochemistry of the livers showed a large increase in the mRNA and protein levels of Cyp1a, which was involved in the biotransformation of A-998679. Induction of CYP1A was confirmed in primary rat, human, and dog hepatocytes. The compound also weakly inhibited CYP1A2 in human liver microsomes. A-998679 activated the aryl hydrocarbon receptor (AhR) in a luciferase gene reporter assay in HepG2 cells, upregulated expression of genes associated with AhR activation in rat liver and enhanced nuclear migration of AhR in HepG2 cells. Collectively these results demonstrate that A-998679 is an AhR activator that induces Cyp1a1 and Cyp1a2 expression, resulting in an autoinduction phenomenon. The unique properties of A-998679, along with its novel structure distinct from classical polycyclic aromatic hydrocarbons (PAHs), may warrant its further evaluation as a tool compound for use in studies involving AhR biology and CYP1A-related mechanisms of drug metabolism and toxicity.

## Introduction

Cytochrome P450 (CYP) induction in the liver complicates the development of new drugs in pharmaceutical industry because of its association with drug–drug interactions and its potential impact on toxicology studies (Coon, 2005; Amacher, 2010). Multiple enzymes and transporters involved in xenobiotic metabolism are inducible through activation of various transcription factors, most notably the aryl hydrocarbon receptor (AhR), the pregnane X receptor (PXR), the peroxisome proliferator-activated receptor (PPARα), and the constitutive androstane receptor (CAR) (Blomme et al., 2009). The activated receptors, along with specific cofactors, localize to their respective DNA response elements, leading to transcription and translation of their target genes, including CYPs, transporters, and phase 2 conjugation enzymes (e.g., Uridine 5′-diphospho-glucuronosyltransferases-UGTs). AhR activation regulates the transcription of CYP1A1 and CYP1A2 and several other genes (Nebert and Dalton, 2006). When AhR encounters a suitable ligand, its chaperone proteins [including heat shock protein 90 (HSP90), p23, and aryl hydrocarbon receptor interacting protein (ARA9)] are displaced, leading to migration to the nucleus where AhR forms a heterodimer with aryl hydrocarbon receptor nuclear translocator (ARNT) with subsequent DNA binding at the dioxin response element (DRE or XRE) and transcriptional activation (Nebert and Dalton, 2006; Barouki et al., 2007). While this may represent the predominant mechanism, data indicate that induction of CYP1A may not be only driven by AhR binding and that more complex cross talk can be involved (Hu et al., 2007).

AhR activation by toxicants, such as 2,3,7,8-Tetrachlorodibenzo-p-dioxin (TCDD), has been associated with multiple undesirable side effects, such as wasting syndrome, chloracne, reactive metabolite formation, mutagenic potential, and other severe toxicities. The direct role of CYP1A in many toxicities remains the subject of investigation and discussion, but it has been clearly associated with reactive metabolite formation, increased oxidative stress, and vascular dysfunction (Kopf et al., 2010; Denison et al., 2011). In the cases of omeprazole and other related drugs considered relatively safe, recent evidence suggests that the toxicity potential resulting from activation of AhR and CYP1A induction is complex and involves multiple factors, including the ligand affinity to AhR, genetics, environmental exposure, pharmacokinetics (PK), or smoking status (Ma and Lu, 2007; Mitchell and Elferink, 2009). For these reasons, the significance of AhR activation in pharmaceutical risk assessment has been the subject of debate.

Increased activity of a specific CYP can result in an increased systemic elimination of a xenobiotic. This phenomenon can complicate the determination of proper clinical dosages and safety margins, can lead to decreased efficacy over time, and increases the likelihood of the formation of reactive oxygen species (Hewitt et al., 2007). CYP3A isoenzymes are most commonly associated with autoinduction (Zhu, 2010). For instance, carbamazepine, dexamethasone, lovastatin, and rifabutin induce expression of CYP3A4 and are also metabolized by the enzyme (Zhu, 2010).

There are fewer reports of CYP1A1 and CYP1A2 involvement with xenobiotic autoinduction, despite its participation in the metabolism of a diverse array of drugs and environmental chemicals (Ma and Lu, 2007). Polycyclic aromatic hydrocarbons (PAHs) are one class of compounds that are both induced and metabolized by the CYP1A subfamily, which in turn can be responsible for the production of several reactive metabolites promoting tumor formation (Barouki and Morel, 2001), but other authors have noted that CYP1A may also have a protective and adaptive role in whole animals (Nebert et al., 2004). However, the most potent inducer of CYP1A, TCDD, is a poor substrate of the enzyme (Ma and Lu, 2007). Omeprazole, a drug that induces CYP1A in humans, is also not metabolized by the enzyme (Diaz et al., 1990; Ma and Lu, 2007).

**Figure 1:**
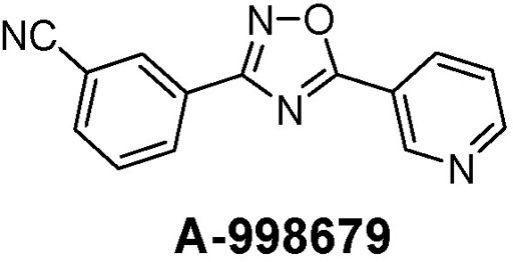
**Chemical structure of A-998679**.

In this report, we describe an experimental activator of AhR resulting in autoinduction through Cyp1a1 and Cyp1a2. A-998679 [3-(5-pyridin-3-yl-1,2,4-oxadiazol-3-yl) benzonitrile] (Figure 1) is an experimental compound that underwent early preclinical characterization for neuropsychiatric indications. It is a novel positive allosteric modulator of α4β2 nicotinic acetylcholine receptors, enhancing the downstream response of these receptors as described previously (Anderson et al., 2009).

In pharmacokinetic studies, repeated dosing was associated with lower plasma exposure over time. The objective of this study was to generate a mechanistic understanding of this phenomenon.

## Materials and methods

### Reagents

A-998679 was synthesized in our laboratories. All reagents, including the probe substrates phenacetin, diclofenac, and dextromethorphan, were obtained from Sigma (St Louis, MO) unless otherwise indicated. Midazolam was purchased from Spectrum Labs (Rancho Dominguez, CA); S-Mephenytoin was from Toronto Research Chemical (Toronto, Canada).

### Rat study

All animal experiments were conducted in accordance with the Guiding Principles in the Use of Animals in Toxicology (http://www.toxicology.org/ai/air/air6.asp) and were approved by Abbott's Institutional Animal Care and Use Committee (IACUC). Male Sprague-Dawley rats [Crl:CD®(SD)IGS BR], weighing ~250 g at study initiation were obtained from Charles River Laboratories, Inc. (Wilmington, MA). Rats were housed singly in ventilated, stainless steel, wire-bottom hanging cages and fed non-certified Rodent Chow (Harlan Labs, Madison, WI), and water *ad libitum* and acclimated for at least 5 days after arrival. Rats were randomly assigned to various treatment groups (3 rats/group) and were dosed once daily by oral gavage with vehicle (0.2% hydroxypropylmethylcellulose at a dose volume of 10 ml/kg) or with 30, 100, or 200 mg/kg of A-998679. All rats were fasted overnight after their last dose, weighed and sacrificed under isoflurane anesthesia. For histopathological evaluation, liver, heart, kidney, spleen, bone (sternum) with marrow, gastrointestinal tract (duodenum with pancreas, jejunum, ileum, cecum, and colon), thymus, and lung were fixed in formalin, routinely processed, and embedded in paraffin. Paraffin sections (4 μm) were stained with hematoxylin and eosin. Liver and small intestine (jejunum) were also flash frozen in liquid nitrogen and stored at −80°C until processing for gene expression profiling. Serum clinical chemistry parameters were quantified using an Aeroset Clinical Chemistry Analyzer (Abbott Laboratories, Abbott Park, IL) and included alanine amino transferase (ALT), aspartate amino transferase (AST), gamma glutamyltransferase (GGT), and alkaline phosphatase (ALP) activities.

### Cell culture

Rat hepatocytes were isolated in a perfusion procedure as described previously (Elliget and Kolaja, 1983; Waring et al., 2001). The beagle dog hepatocytes were isolated in the same manner, except for adjustment of flow rate and buffer volume to scale to liver size. Cells were plated at a seeding density of 1.0 × 10^6^ cells per well on 6-well, collagen-type I coated plates (BD Biosciences, Bedford, MA) in hepatocyte plating medium (Celsis, Baltimore, MD). Cells were allowed to attach for 24 h in a 37°C humidified incubator with a 5% CO_2_ atmosphere and were dosed for 24 h (rat) or 48 h (dog) with vehicle control [0.1% DMSO (v/v)] or 10 μM A-998679 in fresh treatment media (Celsis hepatocyte incubation media) every 24 h. After the last incubation, cells were lysed in 1 ml of Qiazol (Qiagen) and stored at −80°C until RNA isolation. Cryopreserved human hepatocytes (donor designation Hu4198; Invitrogen Life Technologies, Carlsbad, CA) were thawed and plated according to the manufacturer's protocol and allowed to incubate for 24 h. Cells were then dosed with 1, 3, 10 or 30 μM A-998679 for 24 h and harvested as described above. HepG2 cells (American Type Culture Collection, Manassas, VA) were cultured under the same conditions as the hepatocytes using Dulbecco's Modified Eagle's Medium supplemented with 10% fetal calf serum (Invitrogen Life Technologies). The cells were cultured continuously until used in experiments.

### RNA preparation

Frozen liver or jejunum were immediately added to 4 ml of TRIzol reagent (Invitrogen Life Technologies) and homogenized using a Polytron 300D homogenizer (Brinkman Instruments, Westbury, NY). Total RNA was isolated via chloroform extraction and nucleic acid precipitation with isopropanol. Pellets were washed with 75% ethanol and re-suspended in molecular biology grade water. Nucleic acid concentration was determined by OD 260 nm (Smart-Spec, Bio-Rad Laboratories, Hercules, CA), and RNA integrity was evaluated using an Agilent 2100 bioanalyzer (Agilent Technologies, Foster City, CA).

### Microarray hybridization

Microarray hybridization was performed using the standard protocol provided by Affymetrix, Inc. (Santa Clara, CA). Briefly, approximately 5 μg of total RNA was reverse transcribed into cDNA using a Superscript II Double-Strand cDNA synthesis kit (Invitrogen Life Technologies) according to the manufacturer's instructions, except that the primer used for the reverse transcription reaction was a modified T7 primer with 24 thymidines at the 5′ end (Affymetrix). The sequence was 5′-GGCCAGTGAATTGTAATACGAC-TCACTATAGGGAGGCGG-(dT)24-3′. cDNA was purified via filtration (Qiagen, Valencia, CA), phenol/chloroform/isoamylalcohol extraction (Invitrogen Life Technologies) and ethanol precipitation. Purified cDNA was re-suspended in molecular biology grade water, and then used to synthesize biotin-labeled cRNA using the Enzo RNA Transcript Labeling Kit (Enzo Life Sciences, Farmindale, NY) according to the manufacturer's instructions. Labeled cRNA was purified using RNeasy kits (Qiagen), and cRNA concentration and integrity were evaluated. Approximately 200 μg of cRNA was then fragmented in a solution of 40 mM Tris-acetate, pH 8.1, 100 mM KOAc, and 30 mM MgOAc at 94°C for 35 min, and then hybridized to an Affymetrix rat genome RAE230 2.0 array, which contains sequences to roughly 31,000 probe sets, at 45°C overnight using an Affymetrix Hybridization Oven 640. Arrays were subsequently washed, stained twice with strepavidin-phycoerythrin (Molecular Probes, Carlsbad, CA) using the GeneChip® Fluidics Workstation 400 (Affymetrix), and finally scanned using the Affymetrix GeneChip® Scanner 3000.

### Statistics and microarray data analysis

Microarray data were analyzed using Rosetta Resolver software using its error model to detect statistically significant (*p* ≤ 0.05) gene expression changes (version 7.2). The microarray data have been deposited in NCBIs Gene Expression Omnibus and are accessible through GEO Series accession number GSE39525. Data were analyzed through the use of IPA (version 9.0, Ingenuity® Systems, www.ingenuity.com). (Redwood City, CA). Graphpad Prism (version 5.0; Graphpad Software, Inc., San Diego, CA) was used for graphing and statistical analysis, including the standard *t*-test and One-Way ANOVA followed by Tukey's multiple comparison test. A *p*-value ≤ 0.05 was considered significant.

### Pharmacokinetic analysis

Serial blood samples were obtained from a tail vein of each rat 0.5, 1.5, 3, 6, 9, 12, and 24 h after drug administration on the first and last day of dosing. Plasma was separated by centrifugation and stored frozen prior to analysis. A-998679 was separated from the plasma using protein precipitation with acetonitrile. A-998679 and the internal standard were separated from co-extracted contaminants on a 50 × 3 mm Betasil CN column (Thermosystem) with an acetonitrile: 10 mM ammonium acetate mobile phase (35:65, by volume) at a flow rate of 0.4 ml/min at ambient temperature. Concentrations of A-998679 in the plasma extracts were determined by HPLC-MS/MS (API2000; Applied Biosystems). Peak plasma concentrations (*C*_max_) and the time to peak plasma concentration (*T*_max_) were read directly from the plasma concentration data for each rat. A-998679 plasma concentration data were submitted to multi-exponential curve fitting using WinNonlin. The area under the plasma concentration-time curve from 0 to *t* hours (time of the last measurable plasma concentration) after dosing (AUC0-t) was calculated using the linear trapezoidal rule for the plasma concentration-time profiles. The residual area extrapolated to infinity, determined as the final measured plasma concentration (*C*_*t*_) divided by the terminal elimination rate constant (β), was added to AUC0-t to produce the total area under the curve (AUC0-∞).

### Metabolic profiling of A-998679

Identification of the drug metabolizing enzymes (DMEs) of A-998679 was studied in cDNA expressed recombinant enzymes prepared from baculovirus infected insect cells over-expressing rat Cyp isoforms (BD Gentest, Woburn, MA) as listed in Table 2. Incubations were conducted in duplicate using Supersomes. The incubation mixture contained 100 pmol/ml of enzymes, except for Cyp1a1 (200 pmol/ml), 0.1 or 0.21 μM of [^3^H]A-998679 in 100 mM phosphate buffer (pH 7.4). Reactions were initiated with the addition of NADPH at a final concentration of 1 mM and incubated at 37°C for 60 min. At the end of the incubation period, the incubations were terminated by the addition of acetonitrile/ethanol (50/50, v/v) solution. Insect control samples lacking expressed enzyme were used as negative control. The degree of metabolism of A-998679 was assessed by percent of parent remaining using the radio-HPLC method described below.

To assess the involvement of CYP1A2 in the metabolism of A-998679, incubations were conducted in duplicate in human liver microsomes (BD Gentest, Lot # 20567) in the presence or absence of furafylline, a CYP1A2 inhibitor. The incubation mixture contained 0.34 μM [^3^H]A-998679 and 0.5 mg/ml liver microsomal protein in 50 mM phosphate buffer at pH 7.4. Varying concentrations (final concentrations of 0, 0.625, 1.25, 2.5, 5, or 10 mM) of furafylline were co-incubated with [^3^H]A-998679. The reactions were initiated by the addition of 1 mM NADPH and incubated at 37°C for 30 min. The reaction was quenched with acetonitrile and methanol (50/50, v/v). Samples were centrifuged for 10 min at 14,000 rpm (Jouan CR3i centrifuge, Virginia with an AC 2.14 rotor), and the supernatants were analyzed by HPLC with radioflow detection. The degree of inhibition of A-998679 metabolism was determined relative to negative control samples.

Radiolabeled components from the above assays were profiled following separation by HPLC with on line radioflow detector. The system consisted of an Agilent 1100 Series quaternary pump and autosampler connected to a Packard 500TR flow scintillation detector. The data were processed using the Radiomatic™ Flo-one® software v3.65. The scintillant (Perkin Elmer FLO-SCINT™ III) flow was maintained at 3 ml/min. The elution of metabolites was achieved at room temperature on a Luna C18, 5 μm, 100A, 250 × 4.6 mm i.d. column in the presence of a guard column (Phenomenex, Torrance, CA). Mobile phases were A: 25 mM ammonium formate in water with 2.5% methanol (v/v) pH 3.5; and B: acetonitrile; the flow rate was maintained at 1 ml/min. The gradient program began at 0% B at 0 min and was increased linearly to 60% B in 25 min, to 85% B in 5 min, then decreased to 0% B in 2 min.

### RT-PCR

RNA samples were diluted with RNase-free molecular biology grade water to a concentration of 20 ng/μl total RNA. Each sample (2.5 μl) was pipetted in triplicate into a 96-well optical reaction plate (Applied Biosystems, Carlsbad, CA). Reagent mix was made using the TaqMan EZ RT–PCR kit (Applied Biosystems) and 17.5 μl of reagent mix was added to each well. PCR primer and TaqMan probe sequences are listed in Table A1. RT–PCR was performed on the ABI Prism 7900 (Applied Biosystems) with the following parameters: 2 min at 50°C (initial step), 30 min at 60°C (RT), 5 min at 95°C (deactivation), and 40 cycles of 20 s melting at 94°C then 1 min annealing/extending at 62°C. Results were analyzed by calculating the fold change of CYP1A1 and/or CYP1A2 mRNA levels normalized to 28S rRNA (ΔΔ *C*_*t*_) in compound treated cells or tissues compared to vehicle levels. Since Cyp1a1 was undetectable in vehicle livers, an arbitrary *C*_*t*_ = 40 was assigned and used as a basis to calculate fold change.

### Immunohistochemistry

Formalin fixed, paraffin embedded 4 μm sections were rehydrated through 100 and 95% ethanol and deionized water. Endogenous peroxidase activity was quenched by incubating slides in 3% hydrogen peroxide followed by a brief rinse in Tris buffered saline (TBS). No antigen retrieval was used. Sections were blocked with SNIPER (Biocare, Concord, CA) for 5 min and incubated for 1 h with a 1:900 diluted polyclonal rabbit anti-rat Cyp1a1 antibody (Millipore-Chemicon, Temecula, CA). After a TBS wash, the sections were incubated for 30 min with a 1:200 diluted biotinylated goat anti-rabbit secondary antibody (Vector Laboratories, Burlingame, CA). After a brief wash in TBS, the sections were treated for 30 min with ABC reagent (Vector Laboratories), washed in TBS, and then incubated with DAB (3,3′-diaminobenzidine) (Vector Laboratories) for 5 min. Slides were counterstained with hematoxylin and mounted for viewing. All procedures were conducted at room temperature.

### AhR reporter assay

HepG2 cells were transiently transfected overnight with the construct (1200 bp portion of the human CYP1A1 promoter region containing 2 copies of the functional consensus sequence for the DRE in luc2P luciferase vector (SwitchGear Genomics cat# 114555, Menlo Park, CA) using lipofectin (Invitrogen Life Technologies) at 500 ng construct per million cells. Following transfection, the cells were replated in a 96-well format and dosed for 24 h with 0.1% (v/v) DMSO vehicle, 3-MC at 0.05, 0.1, 0.5, 1, 5, 10 μM, and A-998679 at 1.5625, 3.125, 6.25, 12.5, 25, and 50 μM. Luminescence signal was generated using the Dual-Luciferase Reporter Assay System (Promega, Madison, WI) and detected with a Tecan Genios Pro reader (Männedorf, Switzerland).

### Translocation assay

HepG2 cells were plated in Lab-Tek II Chamber slides (Nalgene, Rochester, NY) at a seeding density of 50,000 cells/well. The cells were allowed to attach overnight and were treated for 2 h or 24 h with 10 or 40 μM A-998679, 2 μM 3-methylchloranthrene (3-MC) as a positive control, or 0.1% (v/v) DMSO vehicle. The cells were fixed in fresh 3% paraformaldehyde solution in PBS followed by a PBS wash, and were then permeabilized for 5 min using 0.2% Triton X-100 in PBS followed by another PBS wash. The cells were then incubated with blocking buffer consisting of 1% BSA/PBS solutions and immediately incubated with a 1:50 dilution of rabbit polyclonal antibody to AhR (Abcam, Cambridge, MA catalog# 63636) for 1 h at 37°C. Cells were then washed with PBS, re-blocked for 10 min, and stained with an Alexa 546 donkey anti-rabbit secondary antibody (diluted 1:200; Invitrogen Life Technologies) for 45 min at 37°C. The cells were then washed with PBS, stained with 0.5 μM Hoescht solution for 15 min, and again washed with PBS. The chamber slides were sealed and examined under the microscope. Images were acquired with a SPOT RT SE digital camera (Diagnostic Instruments, Sterling Heights, MI) mounted on a Leica (Wetzlar, Germany) DMIRE inverted florescence microscope using a 20× objective. Image acquisition was done using Metamorph® software (Molecular Devices, Sunnyvale, CA) and percent translocation of the AhR probe was quantitated using MetaXpress (Molecular Devices). Twenty microscope fields were evaluated per well (~3000–6000 cells/well).

### CYP inhibition evaluation

Incubations were performed in human liver microsomes using probe substrate concentrations similar to their corresponding *K*_*m*_ values. Enzyme activity was evaluated in the presence of various concentrations of A-998679. The incubations consisted of the test article at varying concentrations (0–30 μM), probe substrate (concentration ~*K*_*m*_, see Table A2), human liver microsomal protein and 1 mM NADPH in 50 mM phosphate buffer at pH 7.4. The specific incubation conditions for the CYP isoform evaluated are listed in Table A2. Relative quantitation of the metabolites was obtained by LC-MS/MS using an ABSciex (Foster City, CA) API3000 or API4000 Qtrap hybrid linear ion trap mass spectrometer equipped with a TurboIonspray source using either PerkinElmer series 200 pumps or Agilent 1100 series pump with a Leap autosampler. Analytes were separated using a Phenomenex Prodigy ODS(3) 5 μm, 50 × 2.0 mm column at 50°C, eluted and detected under the specific conditions listed in Tables A3, A4, and A5. The analyte to internal standard peak area ratios were determined using Analyst 1.4 software (Applied Biosytems, Carlsbad, CA). The peak area ratios observed in the presence of test article were compared to the vehicle control in order to determine percent activity. To determine the IC_50_, the data were plotted as Log [dose] μM vs. % inhibition, and analyzed with a sigmoidal dose-response (variable slope) fitting equation. The inhibitory responses observed for the positive controls were consistent with historical values.

## Results

### Dosing of rats with A-998679 for 5 days results in a progressive decline in plasma exposure

**Figure 2:**
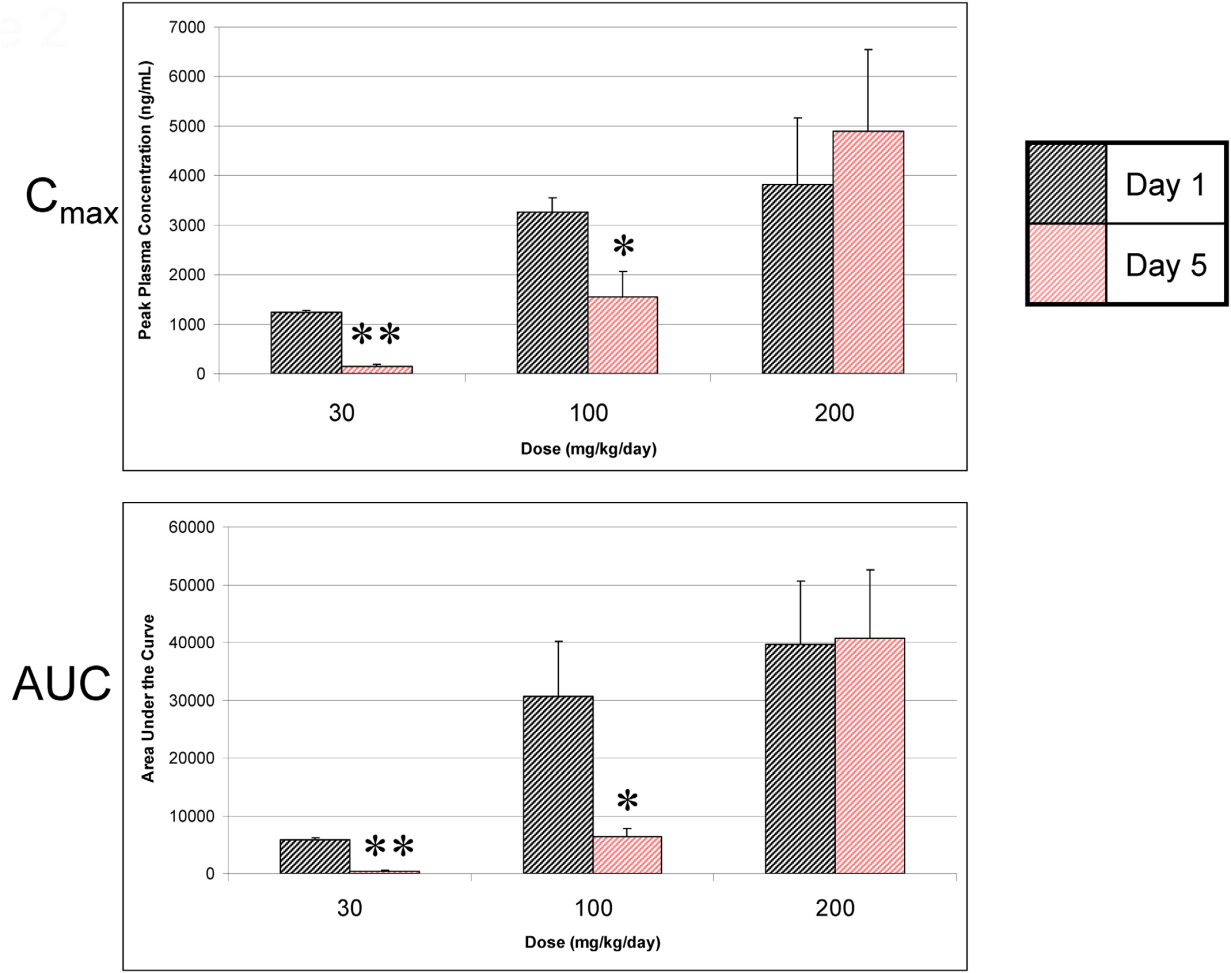
**A-998679 peak plasma concentrations (*C*_max_) and area under the curve (AUC) following oral dosing of rats for 1 or 5 days.** Plasma levels are reported as the mean of 3 rats ± SEM in units of ng/ml (*C*_max_) or ng • h/ml (AUC). The gray bars represent the compound levels on the first day of dosing, and the red bars show the levels on the last day of dosing. The levels drop over time except at the highest dose evaluated. Asterisks indicate that the values were significantly different between day 1 and day 5 (^*^p < 0.05, ^**^p < 0.001).

Dosing of A-998679 in rats for 5 days resulted in a substantial decline in *C*_max_ at the 30 mg/kg (88%) and 100 mg/kg (53%) dose levels (Figure 2) compared to the first day. AUC values were decreased at 30 mg/kg (95%) and 100 mg/kg (80%) after 5 days of dosing. There was a concurrent decrease in **t**_1/2_ and *T*_max_ at these dose levels (data not shown). Interestingly, no change in AUC or *C*_max_ was apparent at the highest dose tested (200 mg/kg). Correspondingly, there was a ~25% increase in relative liver weight in rats treated with 200 mg/kg/day A-998679 but there was no change at the lower doses. No significant changes were noted in clinical chemistry parameters or histopathology.

### A-998679 induces Cyp1a1 and Cyp1a2 in rat liver

**Figure 3:**
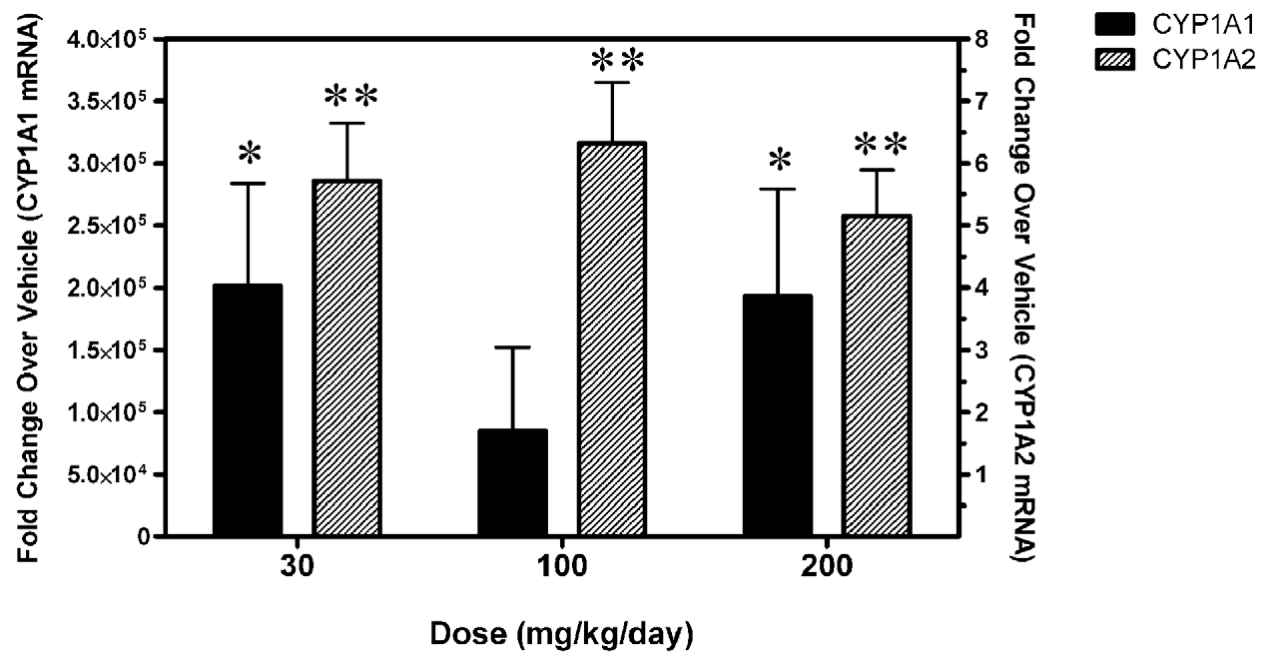
**Levels of Cyp1a1 and Cyp1a2 mRNA in livers from rats dosed for 5 days with 30, 100, or 200 mg/kg/day A-998679 compared to vehicle control animals.** The solid black bars represent Cyp1a1 levels (left y-axis) from an individual rat while the shaded bars represent Cyp1a2 levels (right y-axis). The mRNA data are expressed as fold-change (average ± SD) compared to vehicle. Asterisks indicate that the values were significantly different between treated and vehicle control rats (^*^p < 0.05, ^**^p < 0.001).

**Figure 4:**
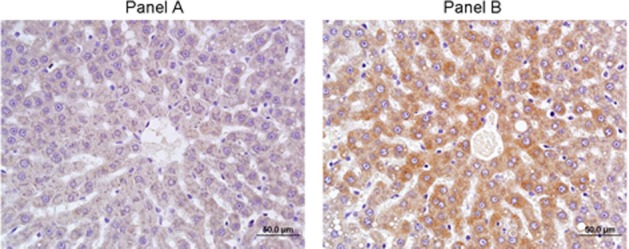
**Immunohistochemistry of Cyp1a1 in rat liver after 5 days of treatment. (A)** Vehicle control. **(B)** 200 mg/kg/day A-998679. Darker areas represent positive protein Cyp1a1 immunostaining.

Microarray analysis was used to profile changes in expression for major Cyp isoenzymes in rat liver after 5 days of dosing with A-998679. Cyp1a1 and Cyp1a2 were increased at the mRNA level in at least one animal starting at the 100 mg/kg dose (~10–100-fold and 2–3-fold, Cyp1a1 and Cyp1a2, respectively). No other major gene expression changes were observed for other Cyp isoforms (data not shown). Using RT-PCR, expression of rat Cyp1a1 and Cyp1a2 mRNA was strongly induced in these livers at all doses (Figure 3). Immunohistochemistry for Cyp1a1 confirmed induction (Figure 4). While Cyp1a1 was not detectable in control livers, immunostaining was observed at moderate levels in centrilobular regions in rats dosed with A-998679. All gene expression changes are provided in the supplementary material to this article.

### A-998679 induces CYP1A1 and CYP1A2 in hepatocytes across several species

**Figure 5:**
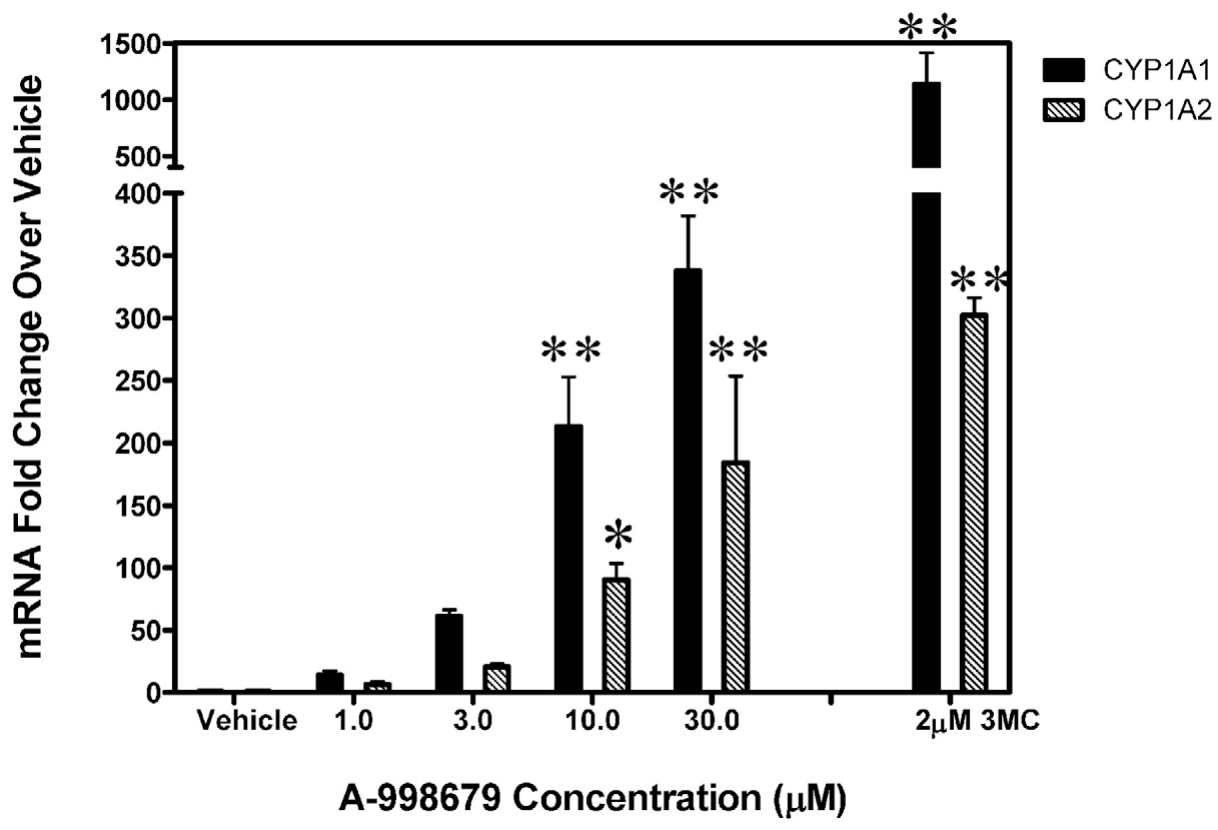
**CYP1A1 and CYP1A2 levels in primary human hepatocytes dosed with increasing levels of A-998679.** The x-axis represents concentration (μM), and the y-axis shows fold-change in CYP1A1 and CYP1A2 mRNA (average ± SD) relative to vehicle control treated cells. 3MC (2 μM) served as a positive control. The black bars represent CYP1A1 and the gray shaded bars indicate CYP1A2. Marked bars indicate that the values were significantly different between treated and vehicle control hepatocytes (^*^p < 0.05, ^**^p < 0.001).

To evaluate whether induction of CYP1A was relevant across species, A-998679 was tested for both CYP1A1 and CYP1A2 mRNA induction in primary human hepatocytes as an *in vitro* model. The compound induced both CYP1A mRNA isoforms in a dose-dependent manner in human hepatocytes starting at levels as low as 3 μM (Figure 5). This result was also observed in primary hepatocytes from rat for Cyp1a1 and dogs for CYP1A2 (data not shown). Thus, A-998679 does not have a species preference with regard to its CYP1A inducing potential in this *in vitro* hepatocyte model.

### Small intestine Cyp1a1 levels are mildly upregulated by A-998679

**Figure 6:**
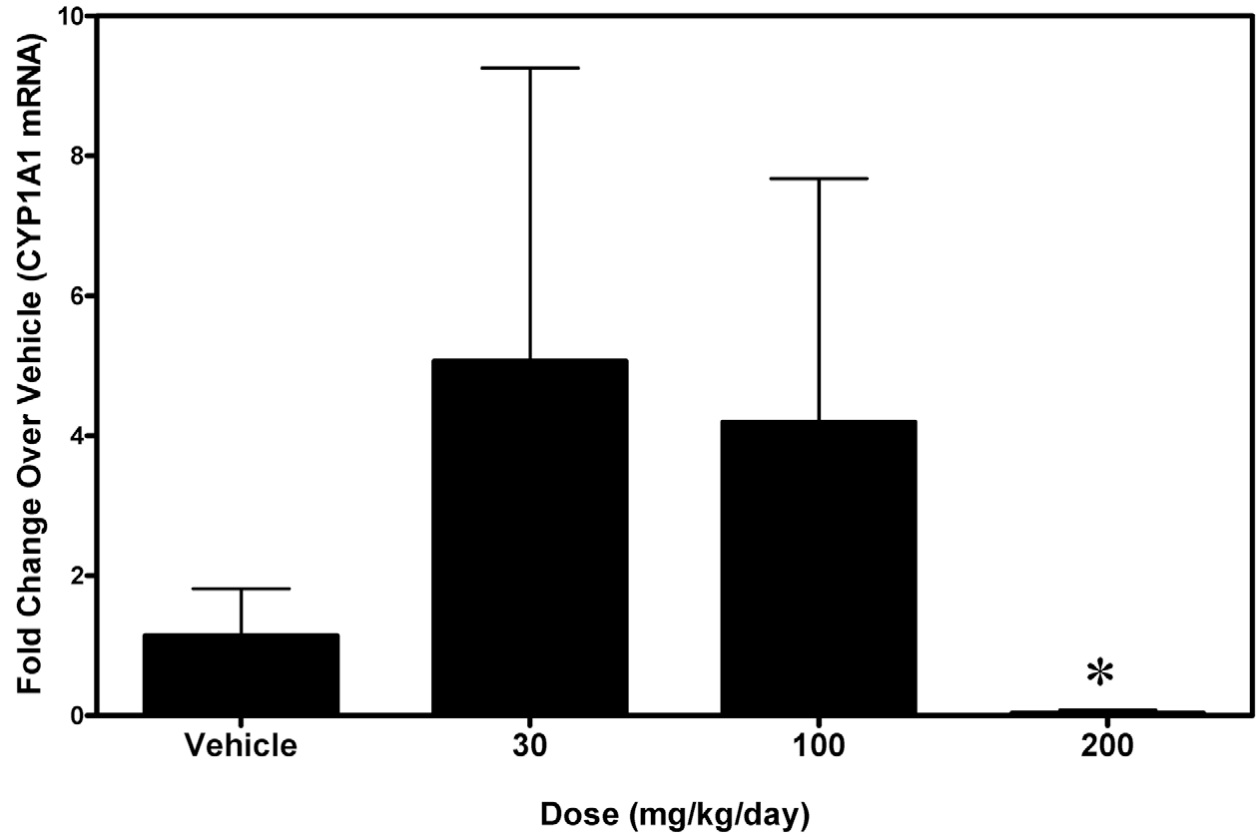
**Levels of Cyp1a1 mRNA in small intestine (jejunum) from rats treated with 30, 100, or 200 mg/kg/day A-998679 compared to vehicle control animals.** The data presentation is the same as described in Figure 3 (average ± SD). Note that at the 200 mg/kg dose, the Cyp1a1 mRNA levels are substantially decreased (below the level of the vehicle controls). Marked bars indicate that the values were significantly different between treated and vehicle control rats (^*^p < 0.05).

Since intestinal DMEs can also affect pharmacokinetic exposure of agents dosed orally, the ability of A-998679 to induce Cyp1a1 in rat jejunum was assessed (Figure 6). At doses of 30 and 100 mg/kg, A-998679 treatment increased mRNA expression of Cyp1a1 with high interindividual variability, albeit at a much lower level than in the liver. Interestingly, at the high dose tested (200 mg/kg), Cyp1a1 mRNA expression was significantly decreased in the jejunum. Levels of jejunum Cyp1a2 mRNA remained unchanged (data not shown).

### A-998679 is an activator of AhR

**Figure 7:**
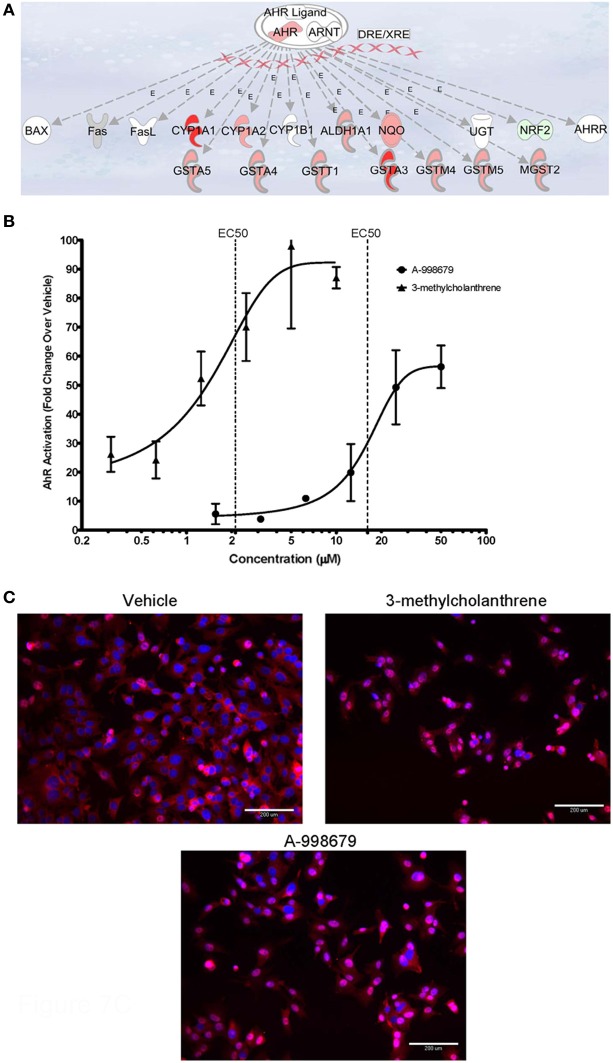
**(A)** Representation of genes induced upon activation of AhR and its subsequent binding to the DRE. Genes displayed as shades of red were increased in mRNA expression, while those in shades of green were decreased. Genes colored in white were not differentially regulated. **(B)** Luciferase gene reporter assay correlating to the degree of AhR activation in HepG2 cells. The concentration of A-998679 or the positive control (3MC) is displayed on the x-axis. Activation of AhR (average fold change ± SD) is indicated on the y-axis. **(C)** Representative fluorescent photomicrograph of the subcellular localization of AhR relative to the nucleus of HepG2 cells after 2 h treatment with vehicle, 3MC (2 μM), or A-998679 (40 μM). AhR is stained pink and the nucleus is stained blue. In the vehicle control, AhR staining was diffuse within the cytoplasm. Upon treatment with 3MC or A-998679, AhR staining intensifies in the nucleus, indicating active nuclear translocation of AhR. The quantitation of nuclear AhR staining from this imaging experiment is depicted in Table 1.

A-998679 was evaluated for its ability to activate AhR. Using rat liver mRNA profiling, the compound upregulated several members of the AhR gene battery, including several glutathione s-transferase (*Gst*) isoforms, NAD(P)H dehydrogenase, quinine 1 (*Nqo1*), aldehyde dehydrogenase (*Aldh*), and *AhR* itself (Figure 7A). Using a luciferase reporter assay with a construct containing a consensus sequence from the human DRE transfected in HepG2 cells, A-998679 activated AhR signaling in a concentration dependent manner (EC_50_ = 16.2 μM) with a maximal increase of 56-fold over vehicle. 3-methylcholanthrene (3MC), a prototypical AhR activator, served as a positive control (EC_50_ = 2.1 μM; 97-fold maximum increase) (Figure 7B). Therefore, 3MC was ~8-fold more potent than A-998679 to activate AhR in this assay. To confirm AhR activation, translocation of AhR from the cytoplasm to the nucleus in HepG2 cells was monitored using fluorescent microscopy (Figure 7C). Both A-998679 at 40 μM and 3MC at 2 μM enhanced AhR nuclear staining relative to the vehicle control after 2 h of treatment (Table 1). The % AhR positive cells dropped substantially after 24 h incubation, but the levels of nuclear AhR remained slightly higher in the 3MC and A-998679 treated cells compared to the vehicle control.

**Table 1:** **Quantitation of AhR nuclear translocation in HepG2 Cells**.

**Compound**	**AhR nuclear staining (% positive cells)**	
	**2 h**	**24 h**
Vehicle control (0.1% DMSO)	15.6	3.4
2 μM 3MC	26.3	11.1
10 μM A-998679	18.8	4.5
40 μM A-998679	29.6	6.3

### Cyp1a1 and Cyp1a2 metabolize A-998679

The ability of a panel of recombinant rat Cyps to metabolize A-998679 was assessed (Table 2). Rat Cyp1a1 and Cyp1a2 were involved in the metabolism of the compound, reducing the percent parent remaining to 0%. Cyp2c11 and Cyp2b1 were also contributors to the metabolism. Other various rat Cyps showed little ability to metabolize the compound.

**Table 2:** **Recombinant rat Cyp phenotype profiling after incubation with [^3^H]A-998679**.

**Cyp isoform**	**% parent remaining and dose**	
	**0.10 μM**	**0.21 μM**
Control	100	100
1A1	0	0
1A2	0	0
2A1	100	100
2A2	90.2	77.8
2B1	87.0	34.5
2C11	78.1	28.8
2C12	100	100
2C13	100	100
2C6	91.0	88.8
2D1	91.9	91.1
2D2	100	98.1
2E1	95.5	91.9
3A1	93.9	90.3
3A2	100	94.2

**Figure 8:**
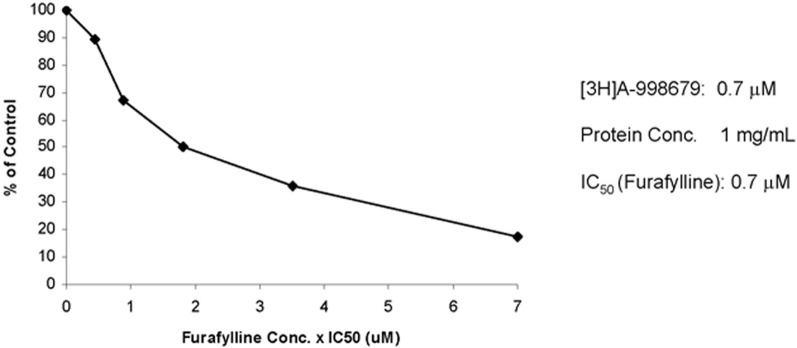
**Effect of furafylline, a specific CYP1A2 inhibitor, on the metabolism of [3H]A-998679 in human liver microsomes.** Incubations with radiolabeled A-998679 were conducted in duplicate in human liver microsomes in the absence or presence of increasing concentrations of furafylline. The degree of metabolism of A-998679 steadily declined as the level of furafylline increased, suggesting a major contribution from CYP1A2.

To further profile the involvement of human CYP1A2 in the biotransformation of A-998679, the metabolic profile was evaluated *in vitro* with human liver microsomes. Furafylline, a specific inhibitor of human CYP1A2, was co-incubated with [^3^H]-A-998679 and metabolism was monitored. As the concentration of furafylline increased (Figure 8), there was a concurrent dose-dependent decline in metabolism of A-998679 (IC_50_ = 0.7 μM). Based on these chemical inhibition data, it is reasonable to conclude Cyp1a2 is a primary contributor to metabolism of the compound; the contribution from other Cyp subfamilies should be minor. Taken together, the data suggest that Cyp1a1 and Cyp1a2 are likely the major routes of metabolism for A-998679 in rats and humans.

### A-998679 inhibits human CYP1A2

The inhibitory effect of A-998679 on the activity of selected CYP isoforms in human liver microsomes was evaluated indirectly by measuring the extent of inhibition of conversion of a probe substrate to a specific metabolite whose formation is primarily mediated by the isoform of interest. A-998679 showed no inhibition of the major CYP enzymes including 2C9, 2C19, 2D6, or 3A4 (IC_50_ > 30 μM), but did inhibit CYP1A2 (IC_50_ = 0.46 μM).

## Discussion

During a 5-day rat exploratory toxicology study, A-998679 dosing resulted in decreased plasma levels and total exposure (AUC) of the compound at 30 and 100 mg/kg/day. Subsequent mechanistic investigation showed that A-998679 is an activator of AhR and an inducer and substrate of Cyp1a. Higher doses (200 mg/kg/day) resulted in sustained levels of the compound, likely due to saturation of metabolism. Hence, the exposure of A-998679 in rats is determined by a delicate balance between induction of Cyp1a at lower levels of the compound and saturation of Cyp1a metabolism at higher levels with a possible enzyme inhibition component (as demonstrated in human liver microsomes). At the 30 mg/kg dose, the plasma concentration was low and enzyme induction dominated the exposure level. As the dose and plasma concentration increased, the metabolism capacity of Cyp1a was overwhelmed and possibly inhibited, which counteracted the effects of induction. This effect results in undesirable pharmacokinetic behavior since the plasma exposure cannot be reliably predicted.

Gene expression profiling showed that, aside from the Cyp1a subfamily, no other drug metabolizing Cyp isoform was induced in A-998679-treated rats. The lack of a dose dependent Cyp1a induction in rat liver is likely due to increased biological variability in whole animals compared to cells and to the fact that the rats were exposed to the compound for 5-days while cells were exposed for 24–48 h. Thus, rat liver had more time to adapt and transcriptionally respond to the presence of the test compound. Since Cyp phenotyping and inhibition experiments revealed that both Cyp1a1 and Cyp1a2 metabolize the compound and since these enzymes were substantially induced, Cyp1a is likely the major contributor to the loss in exposure. Hepatic or intestinal transporters can also influence compound disposition, and A-998679 treatment increased expression of multidrug resistance-associated protein (Mrp3) in liver and Mrp2 in small intestine (data not shown). However, it is not known whether the compound is a substrate of these transporters.

While the autoinduction potential of compounds is not uncommon with the CYP3A family, there are fewer examples of this phenomenon involving CYP1A members. Along with the PAH chemical class, TSU-68, an angiogenesis inhibitor, is an example of a drug candidate that has been linked to autoinduction of CYP1A in humans and rats, resulting in increased clearance (Kitamura et al., 2007, 2008). Other CYP1A inducers, such as TCDD and omeprazole, are not substrates for the enzyme, but nonetheless enhance its expression and activity (Ma and Lu, 2007). While CYP induction is sometimes species-specific as in the case with omeprazole, A-998679 induced CYP1A in rat, dog, and human hepatocytes and/or liver and slightly inhibited the activity of human CYP1A2. Inhibition of CYP1A2 in rats and humans has also been reported with drugs such as fluvoxamine, ciprofloxacin, and oltipraz (Brosen et al., 1993; Langouet et al., 1995; Sofowora et al., 2001; Granfors et al., 2004). The pharmacologic activity of oltipraz, a cancer chemopreventive agent, is thought to partially involve CYP inhibition by preventing the formation of toxic metabolites (Sofowora et al., 2001).

Collectively, our data implicated AhR activation as the mechanism responsible for the decreased plasma exposure of A-998679. Using a gene reporter assay, the compound activated the DRE starting at levels of ~6 μM. Genes downstream of the DRE, including *Cyp1a1* (confirmed at the protein level), *Cyp1a2*, *Nqo1, Aldh*, and *Gst*, were all upregulated in rat liver treated with A-998679. In addition, A-998679 treatment resulted in enhanced translocation of AhR to the nucleus of HepG2 cells. Although no direct AhR-DNA binding assays were conducted, taken together, a weight of evidence approach suggests that direct AhR agonism is the likely explanation for the interesting observations with this compound. Since CYP1A induction was observed in cells or tissues from rat, human, and dog, AhR activation by this compound appears to be species-independent as interpreted from data derived from *in vitro* hepatocyte models.

The toxicological consequence of AhR activation with A-998679 is not known since chronic toxicity studies were not conducted. However, the acute toxicity profile was benign and limited to mild liver weight increase. One potential consequence of AhR activation and subsequent Cyp1a induction is an increase in the overall oxidative stress status of a cell, thus rendering it more susceptible to toxic stimuli (Morel et al., 1999; Barouki and Morel, 2001; Marchand et al., 2004). The toxicological outcome of AhR activation may also relate to the sustainability of the activation (Mitchell and Elferink, 2009). For instance, TCDD is a non-labile and extremely potent activator of AhR which may explain the compound's enhanced toxicity profile. For A-998679 (EC_50_ = 16.2 μM in a DRE cell reporter assay), the potency of AhR activation is several orders of magnitude less than that of TCDD (binding EC_50_ ~pM to nM range) and PAHs (binding EC_50_ ~nM to μM range) (Nguyen and Bradfield, 2008; Mitchell and Elferink, 2009). Rather, the potency of A-998679 is on the same order as some endogenous ligands such as the estrogen ligand, equilenin (reporter assay EC_50_ ~10 μM) (Jinno et al., 2006) and several prostaglandins (reporter assay EC_50_~ 10–20 μM) (Seidel et al., 2001). Although A-998679 requires *in vitro* levels much higher than TCDD or 3MC to elicit its effect, phenotypic changes in rats can be observed with relatively low doses of compound (at least 30 mg/kg with a corresponding plasma *C*_max_ ~ 5 μM).

Most potent AhR activators are planar molecules with the presence of conjugated ring systems or polyhalogenation (Nguyen and Bradfield, 2008). While A-998679 is likely planar in structure (as predicted using ChemDraw 3D), it lacks the other two attributes of classical AhR activators, and thus its lower affinity for AhR is not surprising. Therefore, its mechanism of AhR binding may be more related to atypical AhR activators like the benzimidazoles (e.g., omeprazole), oltipraz, or curcumin (Nguyen and Bradfield, 2008). The former class of compounds does not have the potential to displace [3H]-TCDD in competitive binding assays and are thought to bind AhR outside of the TCDD pocket (Daujat et al., 1992; Lesca et al., 1995). Further investigations will be required to determine whether A-998679 mimics classical methods of binding or if it has a unique mode of interaction.

Cyp1a1 mRNA levels were increased in rat small intestine after treatment with 30 or 100 mg/kg of A-998679, but decreased at the highest dose administered (200 mg/kg). Since Cyp1a is involved in the metabolism of A-998679, its reduced presence may contribute to the lower clearance at high doses. The mechanism behind this repression is unclear. The rat jejunum gene expression profiles and rat and human data from other reports suggest that CYP1A1 (but not 1A2) has basal expression levels much greater than in the liver, and thus enzyme induction could be less apparent (Lin and Lu, 2001; Nishihashi et al., 2006). AhR is expressed in small intestine and can be activated to induce transcription of its target genes (Nishihashi et al., 2006). However, aside from Cyp1a1, mRNAs of genes downstream from the DRE were not induced in rat jejunum samples (data not shown).

AhR function beyond its role as a xenosensor is complex, and the possibility has been raised that some ligands may regulate the effects of AhR diversely between tissue types (Mitchell and Elferink, 2009; Abel and Haarmann-Stemmann, 2010). This may partially explain the repression effect in jejunum with high levels of A-998679, but further investigation will be required for a full understanding. Repression of several Cyp isoforms can be associated with an inflammatory state, but no evidence of inflammation was apparent (Lee et al., 2010). Also, the AhR repressor protein (Ahrr), which inhibits AhR function, is constitutively expressed at higher levels in the small intestine of male rats compared to other tissues (Nishihashi et al., 2006). Consequently, the AhR activation potential of A-998679 may be diminished in small intestine as a result of the heightened presence of Ahrr.

In the present study, the mechanism behind the increased clearance in rats over time of A-998679 was identified. The compound activates AhR, possibly through direct binding and agonism for AhR, with subsequent nuclear translocation and induction of its downstream genes, including Cyp1a. This was identified as a rare autoinduction phenomenon since Cyp1a is involved in the metabolism of the compound. In addition, at higher doses (200 mg/kg/day), the capacity of Cyp1a was saturated which maintained the compound plasma levels. Evidence from human liver microsomes suggests that A-998679 can also act as a weak inhibitor of CYP1A2, which could contribute to higher levels. These interesting molecular properties, along with its novel chemical structure distinct from classical PAHs, may allow for A-998679 to be further investigated as a tool compound in studies involving AhR and CYP biology.

### Conflict of interest statement

The authors declare that the research was conducted in the absence of any commercial or financial relationships that could be construed as a potential conflict of interest.

## Appendix

**Table A1:** **Primer and probe sequences used for RT-PCR assays**.

**Species**	**mRNA**	**Forward primer**	**Probe**	**Reverse primer**
Human	CYP1A1	GAT TGG GCA CAT GCT GAC C	TGG GAA AGA ACC CGC ACC TGG C	CTG TCA AGG ATG AGC CAG CA
	CYP1A2	TGG CTT CTA CAT CCC CAA GAA A	CTG CCA CTG GTT TAC GAA GAC ACA	CCA CAG CTC TGG GTC ATG GT
Rat	Cyp1a1	GCC ATC TGC TGA GGC TCA AC	TCT TCC AAC ATG GGT TTA TGA CAC T	GGT GCT ACA CCC CCA CAT G
	Cyp1a2	TGG CCG TGT TGT CAT TGG	TGT TCC GCC CAG AGC GGT TTC	CAT GAT GAG AAG CAG TGG AAA GA
Dog	CYP1A2	CAA GCG CCG GTG CAT AG	TCT CCC ACT TGG CCA GGA CCT CT	GCA AGA TGG CTA GGA AGA GGA A2
	28S	TTC ACC AAG CGT TGG ATT GTT	TCA CGA CGG TCT AAA CCC AGC TCA CG	TGT CTG AAC CTG CGG TTC CT

**Table A2:** **Incubation conditions used to evaluate the inhibitory effect of A-998679 on specific CYP isoforms**.

**Isoform**	**Probe substrate**		**Substrate concentration (μM)**		**Human liver microsomes [protein] (mg/ml)**	**Incubation (min)**
CYP1A2	Phenacetin		25		0.1	30
CYP2C9	Diclofenac		5		0.05	10
CYP2C19	*S*-Mephenytoin		22		0.2	40
CYP2D6	Dextromethorphan		2.5		0.05	30
CYP3A4	Midazolam		4		0.05	10

**Table A3:** **Mobile phase composition**.

**Isoform**		**Mobile phase A**		**Mobile phase B**
CYP1A2		Water with 0.05% acetic acid		MeOH:ACN (20:80) with 0.05% acetic acid
CYP2C9		Water:MeOH (95:5) with 1mM ammonium acetate		ACN:MeOH (50:50) with 1mM ammonium acetate
CYP2C19		Water:MeOH (95:5) with 1mM ammonium acetate		ACN:MeOH (50:50) with 1mM ammonium acetate
CYP2D6		Water with 0.05% acetic acid		MeOH:ACN (20:80) with 0.05% acetic acid
CYP3A4		Water with 0.1% formic acid		MeOH with 0.% formic acid

**Table A4:** **Elution conditions used for each specific to each analyte**.

**1A2**		**2C9**		**2C19**		**2D6**		**3A4**	
**Time(min)**	**%B**	**Time(min)**	**%B**	**Time(min)**	**%B**	**Time(min)**	**%B**	**Time(min)**	**%B**
0	0	0	15	0	10	0	5	0	20
0.5	0	0.5	15	0.5	10	0.1	5	0.5	20
0.6	40	1.5	95	2.2	100	2	60	3	100
2.4	40	2.5	95	2.9	100	2.1	100	3.1	20
2.5	100	2.6	15	3.0	10	2.7	100	3.6	20
3.0	100	3.0	15	3.5	10	2.8	5	−	−
3.1	0	−	−	−	−	3.4	5	−	−
3.5	0	−	−	−	−	−	−	−	−
Flow rate: 0.5 ml/min		Flow rate: 0.45 ml/min		Flow rate: 0.65 ml/min		Flow rate: 0.7 ml/min		Flow rate: 0.5 ml/min	
for 2.4 min, followed									
by 0.7 ml/min									

**Table A5:** **Specific multiple reaction monitoring (MRM) transitions monitored to quantify the analyte of interest were as follows**.

**Isoform**	**Metabolite**		**Metabolite MRM**		**Internal standard (IS)**	**IS MRM**	**Ionization mode**
CYP1A2	Acetaminophen		152 → 110		Acetanilide	136 → 94	Positive
CYP2C9	4-hydroxy diclofenac		310 → 266		Mefenamic acid	240 → 196	Negative
CYP2C19	4-hydroxy mephenytoin		233 → 190		Diphenyl hydantoin	251 → 102	Negative
CYP2D6	Dextrorphan		258 → 157		Levallorphan	284 → 157	Positive
CYP3A4	1-hydroxy midazolam		342 → 203		α-hydroxytriazolam	359 → 176	Positive
